# Is caregiving by baby boomer women related to the presence of depressive symptoms? Evidence from eight national surveys

**DOI:** 10.1186/s12905-018-0696-8

**Published:** 2018-12-19

**Authors:** Chi Chiao, Yun-Yu Chen

**Affiliations:** Institute of Health and Welfare Policy and Institute of Public Health, School of Medicine, National Yang-Ming University, No. 155, Sec. 2, Li-Nong St., 112 Taipei, Taiwan, Republic of China

**Keywords:** MeSH, Depressive symptoms, Boomer women, Caregiving, Social status, Population-based survey

## Abstract

**Background:**

A common hypothesis is that caregiving is deleterious to women’s mental health. International studies continue to emphasize the importance of mental health issues for women. Yet only a few researchers have used population-based surveys to explore the association between caregiving and depressive symptomatology in the context of the community, and even less is known about this aspect of Baby Boomer women in a global context.

**Methods:**

The present study uses eight international surveys covering nineteen nations (*N* = 15,100) and uses multilevel logistic models to examine possible linkages between caregiving and the likelihood of depressive symptoms among Baby Boomer women, when taking individual-level and country-level social factors into consideration.

**Results:**

The various analyses found a significant variation in the likelihood of depressive symptoms among these Boomer women across the nations investigated and across both individual-level and country-level characteristics. The significant association of caregiving by women and the likelihood of depressive symptoms is related to their social status in some nations (OR = 1.30; *p* < 0.001). Boomer women living in countries with high rates of female participation in managerial/professional work (OR = 1.04; *p* < 0.05) and living in countries where women are often in vulnerable employment (OR = 1.01; *p* < 0.05) are at greater risk of depressive symptomatology.

**Conclusions:**

These findings demonstrate that the depressive consequences of caregiving by women are, to some degree, contingent upon social context and structure. Policies aimed at promoting mental health among female Baby Boomers should therefore be context specific.

## Background

Despite international support for improving mental health over the past few decades, the association between health and caregiving remain a concern, particularly among women of low social status in general, and among those living in social disadvantaged countries in particular [1, 2]. Drentea (2007) defined caregiving as *the act of providing unpaid assistance and support to family members or acquaintances who have physical, psychological, or developmental needs* [3]. Women, across a wide range of cultures and countries consistently inherit primary responsibility for such care. Prior studies have suggested that those responsible for caregiving are more psychologically distressed than non-caregivers across a number of different contexts and situations [2, 4]. When viewed from a life course perspective, the amount of psychological distress generated by caregiving, if any, may be a function of the amount of time spent giving care, as well as being related to the caregiver’s gender, age, race, education, experience, and relationship with the care receiver [5, 6]. Providing care for others disrupts one’s personal life and creates additional worries, which can result in a higher level of psychological distress [7, 8], although the strength of that relationship is known to differ across various demographic groups [9]. However, it should be noted that such a relationship was found to be absent when the act of caregiving itself and the level of psychological distress of both Baby Boomers and their predecessors were analyzed [9].

Baby Boomers are a special social group and the name refers to the generation of individuals born between 1946 and 1964 who experienced the rapid global revival that occurred after World War II. Given their higher levels of education and consequent greater employment in professional and managerial positions, these Baby Boomers are markedly different from members of their preceding generation [10–12]. Living in a far more prosperous, peaceful and diverse environment, these Baby Boomers experienced a higher standard of living, better access to medical care, and little, if any, fear of war. Despite that environment, and while mortality rates were indeed lower [13], studies have shown these Baby Boomers were more likely than pre-Baby Boomers to self-report poorer health [14] and higher levels of psychological distress [15].

In particular, female Baby Boomers have been found to be considerably different from their predecessors in their approach to caregiving [16]. Given their higher level of education, increased participation in the labor force, and the greater importance of their own career, female Baby Boomers, especially in mid-career, looked for other alternatives in order to fulfill the caregiving needs of their old-aged parents (nursing homes, assisted living, etc.) and new-born offspring (daycare centers) [17]. In short, female Baby Boomers have sought to balance personal self-achievement and family care as their economic and technological evolution has developed.

These generational changes seem to occur to a varying degree within neighborhoods, organizations and communities, as well as across nations, with public policy, cultural norms and values, and the surrounding social environment all having some relationship to the physical and mental health of these Baby Boomers [18]. In a study across 53 nations conducted by the World Health organization (WHO), approximately one-seventh of the variance in depression-related scores was significantly accounted for by country-level differences in socio-economic status and income inequality, although at the same time individual-level factors accounted for approximately six-sevenths of the variance [19]. An earlier preliminary study by Pickett et al. (2006) also found that there were strong positive linear associations between the prevalence of mental illness and income inequality (*r* = 0.73) based on data from eight nations [20].

Within and across nations and cultures, income inequality has been linked to the relative social status of women; this makes gender inequality, in all of its dimensions, an important variable when discussing the relationship between caregiving and depressive symptomatology. Prior research has suggested that women employed in managerial and professional positions, with higher earnings, greater economic and reproductive autonomy, and greater levels of political participation, have a higher social status and, more importantly, experience less depressive symptomatology [21–23]. However, it is against this background that female Baby Boomers seek to balance activities related to self-achievement and family care to a far greater extent than their predecessors. Therefore, the present study sought to bridge the aforementioned knowledge gaps related to the inconsistent results from prior studies regarding the relationship between caregiving and depressive symptoms at an individual level. Furthermore, in the context of the association between caregiving and depressive symptoms, we further examined the degree to which Baby Boomers psychologically benefit from their higher levels of education and changing social role,s as well as examining the extent to which social status and roles are contingent upon national context or culture.

## Methods

### Data

A search for global aging data was carried out and information were obtained from all possible public-access surveys that included Baby Boomer cohorts and the various major explanatory variables of interest to the present investigation. This yielded eight international datasets, including the 2003 Taiwan Longitudinal Study of Aging (TLSA; participation rate: 79%), the 2006 Korean Longitudinal Study of Aging (KLoSA; participation rate: 75%), the 2002 English Longitudinal Study of Aging (ELSA; participation rate: 67%), the 2003 Health and Retirement Survey (HRS; participation rate: 86%), the 2004 Survey of Health, Aging and Retirement in Europe (SHARE; participation rate: 62%), the 2007 Japanese Study of Aging and Retirement (JSTAR; participation rate: 60%), the 2011 China Health and Retirement Longitudinal Study (CHARLS; participation rate: 81%) and the 2001 Mexican Health and Aging Study (MHAS; participation rate: 92%). Informed consents relating to the aforementioned surveys were given in writing by each respondent at the start of the interviews. All data was anonymized before use.

The TLSA is a nationally representative survey that was conducted over 3–4 years in Taiwan involving respondents who were aged 50 years old and above, although only data from the supplemental sample recruited in 2003 was analyzed as part of this study. KLoSA, JSTAR, ELSA, CHARLS, MHAS and HRS are nationally representative surveys conducted in Korea, Japan, UK, China, Mexico and the US over 2-year periods targeting individuals 45-years old or more and their spouses. When using the HRS, only the cohort “Early Baby Boomers”, whose first survey was conducted in 2004, was included in the study. SHARE provided a dataset that included people ages 50 and above and their spouses for each of twelve European countries: Austria, Belgium, Germany, Denmark, France, Greece, Israel, Italy, Netherlands, Spain, Sweden and Switzerland.

This study analysis was restricted to 16,733 female Baby Boomers born in 1946–1954 from nineteen countries. For all but the TLSA, KLoSA and MHAS, we used harmonized datasets, which allowed the possibility of combining variables of interest from the heterogeneous sources into an integrated form and information on the harmonized data can be found at www.g2aging.org. All datasets are publicly available for research purposes and the study protocol for using these databases was approved by their appropriate institutional review boards and the Research Ethics Committee of National Yang-Ming University (Taipei, Taiwan).

### Measurement of depressive symptoms and caregiving

*Depressive symptoms* were assessed by means of various versions of the Center for Epidemiologic Studies Depression scale (CES-D) [24]. In order to compare the data across the various nations, we dichotomized the data into two categories: depressed or not depressed, using cut-points that had been validated for each of the scales (see Appendix A) [25–31].

*Caregiving* was defined by whether or not personal care for others (i.e., bathing, clothing and washing) was provided by the individual. Given that different surveys were being used, the questions used to assess caregiving were not exactly the same across the datasets. HRS, JSTAR, MHAS, KLoSA, TLSA and SHARE specifically assessed whether personal care had been provided to others. However, there were some inconsistencies. Furthermore, the operational definition of caregiving in ELSA and CHARLS was slightly different to that of the surveys mentioned above. In ELSA, “Did you look after anyone in the past week (including your partner or other people in your household)?” was used. According to the project’s instructions, “look after” could represent looking after grandchildren after school, babysitting etc., as well as caring for sick or disabled friends or relatives (ELSA, 2002). In CHARLS, caregiving was indexed by answers to the following question: “Did you or your spouse take care of your parents or parents-in-law during the last year in assisting them in their daily activities or other activities (e.g., household chores, meal preparation, laundry, going out, grocery shopping, financial management, etc.)?” The question addressed instrumental care and served in the present study as a proxy for measuring personal care.

### Individual-level factors

Educational level, marital status and job status were included as individual level control variables in this study. Educational level was determined using the International Standard Classification of Education 1997 code (ISCED97), developed by the United Nations Educational, Scientific and Cultural Organization (UNESCO), and this has been previously employed to assess standard concepts, definitions and classifications related to educational levels across countries. Seven levels are specified as level 0 - pre-primary education, level 1 - primary education, level 2 - lower secondary education, level 3 - secondary education, level 4 - post-secondary non-tertiary education, level 5 - first stage of tertiary education, and level 6 - s stage of tertiary education. To accommodate inconsistencies in the distribution of educational level across different countries, educational level was converted to an ordinal scale, with larger numbers indicating higher educational levels.

Marital status was assessed by the question “What is your current marital status?” for TLSA, CHARLS and MHAS. Otherwise, current marital status was drawn from harmonized HRS, harmonized SHARE, harmonized JSTAR, harmonized KLoSA and harmonized ELSA to indicate whether the respondent was partnered/married or single (never married, divorced, separated and widowed included).

Work status was measured dichotomously in HRS, SHARE, JSTAR, KLoSA, ELSA, TLSA, and MHAS to indicate whether or not the respondents are currently employed. In CHARLS approximately half of the Chinese respondents reported that they worked as a farmer. Three questions (“Did you engage in agricultural work for more than 10 days in the past year?”, “Did you work for at least one hour last week?”, and “Do you have a job but are temporarily laid-off, or on sick or other leave, or in-job training?”) were used to determine the respondent’s work status. People who answered “yes” to any of the questions were regarded as being currently employed.

### Country-level factors

In this study, we are focused mainly on the association between women’s caregiving and depressive symptoms in the context of their social status and structure. Several indicators describing women’s life with respect to reproductive health, employment, and political participation were utilized to reflect their gender-related social status/role within each of the countries.

Adolescent fertility (AF) was used to measure women’s reproductive rights, and was indexed by births per 1000 women ages 15–19. Even though AF has been criticized for its high correlation with GDP and having no gender-specific counterpart for comparison [32], Williams (2013) found it to be a reasonable way to assess gender inequality, with higher rates of adolescent pregnancy suggesting greater gender inequality within a country [33]. Furthermore, early pregnancy may well have a deleterious effect on women’s health and personal achievement and thereby prevent women from accumulating human capital and elevating their social status [34].

Employment at the country-level was indexed in two ways: (1) percentage of women employed in managerial or professional occupations. A higher percentage of women in such occupations has been associated with a higher level of education and better promotion opportunities for women [35]; and (2) the female labor force participation rate (FLFPR), which refers to the proportion of females, age 15 and older, who are economically active (including both those employed and those unemployed but intending to find a job). Additionally, given that resources invested and products produced in the informal sector of an economy are typically overlooked in official statistics [36], we also chose to include a measure of women’s participation in so-called vulnerable work within each country. This statistic, defined by the UN as the percentage of women serving as unpaid family workers or self-employed workers in relation to total female employment within a country, and this statistic has also been adopted to examine women’s status in the informal sector [32, 36].

Finally, the proportion of seats held by women in national parliaments was used as an indicator of female political participation at the country level in this study. Prior research has shown this to be associated with the mortality rate of men and women [21, 23]. Country-level data was obtained from the World Bank Open Data (http://data.worldbank.org/; see Table 1 for details).

**Table 1:** Summary statistics of the surveys’ datasets

Dataset (year)	Country	N	Age (Mean, Std Dev)	Prevalence of depressive symptoms (%)	Prevalence of caregiving status (%)	Proportion of women in managerial or professional occupations	Female vulnerable employment rate	Proportion of seats held by women in national parliaments	Adolescent fertility rate	Female labor force participation rate
CHARLS (2011)	People’s Republic of China	2537	60.55 (2.51)	47.34	9.10	44	71^a^	20	8	67
JSTAR (2007)	Japan	561	56.98 (2.58)	19.61	26.03	40	14	9	5	48
KLoSA (2006)	Korea	1349	56.00 (2.56)	14.31	3.57	35	29	13	2	50
MHAS (2001)	Mexico	2642	51.78 (2.29)	37.65	9.70	40	33	24	71	41
TLSA (2003)	Taiwan	774	53.37 (2.04)	10.98	4.93	41^b^	20^b^	30^c^	8^d^	48^b^
ELSA (2002)	UK	1770	52.90 (2.26)	25.54	24.66	42	6	20	26	55
HRS (2004)	USA	1365	52.98 (1.80)	18.97	7.13	50	6^e,f^	15	41	58
SHARE (2004)	Austria	236	54.46 (2.61)	22.88	15.02	45	8	34	12	51
	Belgium	697	54.00 (2.57)	29.56	13.36	44	10	35	11	46
	Denmark	287	54.15 (2.59)	24.74	8.83	48	4	37	6	61
	France	594	53.96 (2.61)	39.56	9.55	46	5	12	10	50
	Germany	506	53.83 (2.36)	19.96	10.24	48	7	32	11	51
	Greece	510	53.70 (2.51)	29.80	9.15	41	28	13	11	42
	Israel	562	53.98 (2.57)	33.27	13.08	48	5	15	15	50
	Italy	432	54.50 (2.52)	36.34	6.84	43	17	12	7	38
	Netherlands	603	54.27 (2.52)	21.56	9.76	43	8	37	6	57
	Spain	394	53.80 (2.51)	35.79	4.70	44	11	36	12	46
	Sweden	524	54.27 (2.55)	25.19	10.72	49	4	45	6	59
	Switzerland	162	53.75 (2.58)	28.40	7.64	43	10	25	5	60

*Note*: ^a^China Labour Statistical Yearbook 2006 ^b^Directorate General of Budget, Accounting and Statistics (DGBAS) (http://www.dgbas.gov.tw/) ^c^The Legislative Yuan of Republic of China (http://www.ly.gov.tw/) ^d^ Department of Household Registration Affairs (http://www.moi.gov.tw/stat/) ^e^LABORSTA (http://www.ilo.org/ilostat/) ^f^Employer, own account worker and unpaid family worker were included

### Analytical strategy

In order to extract interferences related to cohort effects, we focused only on female Baby Boomers born during the early years of the period in question for this study (*N* = 16,733). Our analytical sample was further restricted to respondents born between 1946 and 1954 and yielded a total sample of 15,100 individuals who had provided complete responses on major measurements. Our analysis began with descriptive analyses that characterized the prevalence of depressive symptomatology among Boomer women for each of countries. Given that women within a given country and participating in the same survey often experience common contextual and data collection influences and therefore their subsequent reports regarding depressive symptom patterns are more likely to be similar than those of women in other countries participating in related but somewhat different surveys. We then corrected the estimated standard errors of coefficients for this kind of clustering using Stata 14.0 [37], which had been updated with the *gllamm* program for multilevel models [38]. This process also allowed us to estimate the random effect within our data.

We hypothesized that individual characteristics and women’s status at the country level were jointly associated with individual depressive symptomatology. In order to take survey sampling into consideration, we used a 3-level logistic regression approach for the analysis [39]. Individual respondents were studied at level 1 and this was nested within a level-2 analysis of the countries, which again was nested within a level-3 analysis of the complete survey data. The dataset at each level of the analysis was examined as a pooled analytical sample that contained the eight international datasets. To assess whether the likelihood of depressive symptoms varied across countries, the gross variance in the likelihood of depressive symptoms associated with countries was first estimated using a null model that did not include explanatory variables involving random variation within and between individuals, countries, and surveys, that is an intercept and error term [39]. The null model provided the basis for decomposing the total variance for the likelihood of depressive symptoms into the sum of individual-level and country-level variances. Using this 3-level random coefficient logistic regression approach, intra-country correlation was approximated by σ_c_^2^ + σ_s_^2^ /(σ_c_^2^ + σ_s_^2^ + σ_e_^2^), where σ_c_^2^ denotes country-level variance, σ_s_^2^ denotes survey-level variance, and σ_e_^2^ denotes individual-level variance, which was set to π^2^/3 (equal to 3.29) [38, 39]. The random effect at the country level was found to be significant (*p* < 0.05), and the intra-class correlation coefficient was 0.11. Based on these findings, multilevel logistic regression was employed in the present study.

We progressively elaborated the significance of individual caregiving factors in relation to the likelihood of depressive symptomatology. In Model 1, we explored the individual-level relationship between caregiving and the likelihood of depressive symptoms by controlling for other social role factors. We included an interaction term for marital status with employment in order to explore the possible effects of multiple social roles in depressive symptoms. Then, we further examined whether the social status of women at the country level accounts for variation in the likelihood of depressive symptoms among countries. Models 2–6 then included individual-level factors and thereby expanded our analysis by adding each of indicators of women status at the country level to address the contribution of these indicators to variation in the likelihood of depressive symptomatology. This one-by-one progressive strategy is due to concerns about the statistical power of the sample sizes of the nineteen countries included in the analysis. The results obtained from all models are presented as odds ratios (OR) with 95% confidence intervals (CIs). Variables used in regression models may be highly correlated. We employed analyses of the variance inflation factor (VIF) and tolerance to assess multicollinearity. VIFs were less than 10 and tolerance values were greater than 0.1, which means that no regard needs to be paid to multicollinearity concerns. Finally, in terms of our approach to the analysis, it was found that Models 1–6 also represent a significant improvement in fit over the null model without covariates.

## Results

Table 1 presents the prevalence of depressive symptoms across individual countries and summarizes the statistics for the country-level indicators used in our analyses. The depressive prevalence in Baby Boomer women ranged from 20 to 40% in European countries, was 19% in the U.S., and ranged from 11 to 47% in Asian countries. Table 2 presents the 3-level multilevel models that sequentially elaborate on the relationships between caregiving at the individual level, women’s status at the country level, and the likelihood of depressive symptomatology. The null model indicates that there was significant variation in the likelihood of depressive symptoms among the various countries. The intra-class correlation (ρ) is 0.11, indicating that 11% of the variation in the likelihood of symptoms is at the country-level (the results not tabled).

**Table 2:** Multilevel regression of the likelihoods of depressive symptoms in relation to caregiving and social risk factors at individual and country levels among female Baby Boomers

	OR (95% CI)					
	Model 1	Model 2	Model 3	Model 4	Model 5	Model 6
*Individual-level variables*						
Caregiving	1.30 ***	1.30 ***	1.30 ***	1.30 ***	1.30 ***	1.30 ***
	(1.16–1.46)	(1.16–1.46)	(1.16–1.46)	(1.16–1.46)	(1.16–1.46)	(1.16–1.46)
Married	0.46 ***	0.46 ***	0.46 ***	0.46 ***	0.46 ***	0.46 ***
	(0.40–0.52)	(0.40–0.52)	(0.40–0.52)	(0.40–0.52)	(0.40–0.52)	(0.40–0.52)
Employed	0.53 ***	0.55 ***	0.55 ***	0.55 ***	0.55 ***	0.55 ***
	(0.47–0.65)	(0.47–0.64)	(0.47–0.65)	(0.47–0.65)	(0.47–0.65)	(0.47–0.65)
Married x Employed	1.46 ***	1.46 ***	1.46 ***	1.46 ***	1.46 ***	1.46 ***
	(1.23–1.74)	(1.23–1.74)	(1.23–1.74)	(1.23–1.74)	(1.23–1.74)	(1.23–1.74)
Education	0.78 ***	0.78 ***	0.78 ***	0.78 ***	0.78 ***	0.78 ***
	(0.76–0.81)	(0.76–0.80)	(0.76–0.81)	(0.76–0.81)	(0.76–0.81)	(0.76–0.81)
Age	0.98 *	0.98 *	0.98 *	0.98 *	0.98 *	0.98 *
	(0.97–0.998)	(0.97–0.999)	(0.97–0.997)	(0.97–0.997)	(0.97–0.998)	(0.97–0.998)
*Country-level variables*						
Proportion of women in managerial or professional occupations		1.04 *				
		(1.01–1.08)				
Female vulnerable employment			1.01 *			
			(1.0001–1.01)			
Proportion of seats held by women in national parliaments				0.99		
				(0.98–1.00)		
Adolescent fertility					1.01	
					(0.99–1.02)	
Female labor force participation						1.00
						(0.98–1.01)
*Random variance component*	Est. (S.E.)	Est. (S.E.)	Est. (S.E.)	Est. (S.E.)	Est. (S.E.)	Est. (S.E.)
Intercept at level 2	0.06 (0.03)*	0.30 (0.06)*	0.10 (0.04)*	0.08 (0.04)*	0.06 (0.03)*	0.10 (0.03)*
Intercept at level 3	0.39 (0.13)***	0.00002 (0.001)	0.19 (0.09)*	0.32 (0.08)*	0.30 (0.15)*	0.22 (0.09)*
*Comparison to null model*						
Chi-square	264.84***	261.94***	264.43***	262.96***	265.50***	264.30***
Degrees of freedom	6	7	7	7	7	7

* *p* < 0.05; **p < 0.01; ***p < 0.001

*Abbreviations*: *CI* confidence interval, *OR* odds ratio

Analysis using Model 1 shows that the coefficients for providing care are positive and significant; that is, women who provide caregiving were 1.30 times more likely to report depressive symptoms than their counterparts. Model 1 also includes marital status, employment status, and their multiplicative interaction terms as a set. As shown, married and employed women tend to experience depressive symptoms less often than women who are not currently married (OR = 0.46; *p* < 0.001) or are unemployed (OR = 0.53; *p* < 0.001). The coefficient for the interaction between marital status and employment status is significant with the likelihood of depressive symptomatology being lower among married women, being lower among employed women, and yet being significantly increased among women who are both married and employed (OR = 1.46; *p* < 0.001). The greatest difference in the likelihood of depressive symptoms is between women who are both married and employed vs. women who are neither married nor employed. In addition, the odds of depressive symptomatology decreases as age increases (OR = 0.98; *p* < 0.05) and the odds also decrease with an increased in level of education attainment (OR = 0.78; *p* < 0.001).

Models 2–6 add the main effects of the five country-level variables with respect to women’s status: proportion of women employed in managerial or professional occupations, female vulnerable employment, proportion of seats held by women in national parliaments, adolescent fertility, and female labor force participation. Compared to Model 1, the coefficient for caregiving remains unchanged, indicating the caregiving effect is independent of the country-level indicators. As shown in Table 2, the odds of depressive symptomatology were increased by the number of women employed in managerial or professional occupations increased (OR = 1.04; *p* < 0.05) and also increased as female vulnerable employment increased (OR = 1.01; *p* < 0.05). In contrast, depressive symptomatology showed no significant association with the other three indicators, namely the proportion of seats held by women in national parliaments, adolescent fertility, and female labor force participation. As shown at the bottom of Table 2, each of the models represents a significant improvement in fit over the null model.

## Discussion

The results of this study suggest: (1) there is substantial variation in the likelihood of depressive symptomatology among Baby Boomer women when examined with respect to caregiving and social status; (2) caregiving is associated with higher levels of depressive symptomatology in Baby Boomer women even when other social factors are controlled for at both the individual and country levels; (3) the likelihood of depressive symptomatology does vary significantly across countries; and (4) the proportion of women employed in the non-agricultural sector and the vulnerable employment rate at the country level are associated with the likelihood of depressive symptomatology over and above individual social status.

Caregiving was found to have a significant association with the likelihood of depressive symptomatology across countries, showing the important effect that caregiving has on depressive symptomatology among Baby Boomer women. This result is consistent with prior findings [2, 3] and expands our understanding of caregiving among early Baby Boomers at both the individual level and within a particular nation. Even when other factors related to social role and women’s status indicators at the country-level have been adjusted for, caregiving is still significantly associated with the likelihood of experiencing depressive symptomatology. The findings once again suggest that caregiving should be taken into consideration when seeking to understand the relationships between social roles, country context, and depressive symptomatology.

However, inconsistent with prior studies [21–23], we found that both a higher percentage of women employed in managerial or professional occupations and a higher percentage of women involved in vulnerable employment were associated with a higher odds of depressive symptoms among female Baby Boomers; this is despite female labor force participation not showing a significant association with depressive symptomatology. Our findings further demonstrate that women in the countries with a higher proportion of women employed in managerial or professional occupations are more likely to report depressive symptoms than their counterparts with lower proportions of women employed in managerial or professional occupations. One possible explanation for the association between women’s employment and depressive symptoms among the Baby Boomer women is that these Baby Boomer women with multiple social roles may be more likely to be depressed due to experiencing conflict between work and family compared to women without such role stress. Similarly, a higher rate of female vulnerable employment means that a major employment for women in that country is an insecure job, such as a market vendor, being self-employed or acting as unpaid family labor. These types of job provide low or no pay with few or even no work benefits in countries such as the People’s Republic of China. Our results demonstrate that Baby Boomer women who live in countries with a higher rate of female vulnerable employment are more likely to report depressive symptoms. To our knowledge, this is the first study that has explored the relationship between country-level female employment status and depressive symptomatology.

To further explore the effect of caregiving on depressive symptomatology with respect to women employment status at a country level (Table 3), additional analysis was conducted by grouping the nineteen countries into *higher* and *lower* status groupings for women’s employment. We found that the caregiving effects are significant among the countries with a *higher* status, namely having a higher proportion of women employed in managerial or professional occupation, having a lower percent of female vulnerable employment, and having a higher rate of labor force participation. These countries included the United States, Israel, and various countries in western and northern Europe. In contrast, caregiving was found not be a significant factor among the countries with a *lower* women’s employment status (e.g., southern Europe, East Asia, and Mexico). These findings suggest that Baby Boomer women with a *higher* employment status seem to be more likely to report a harmful caregiving effect and that a significant interaction between employment and marital status can also be found for these Baby Boomer women.

**Table 3:** Results from the multivariate logistic regression models of the likelihoods of depressive symptoms stratified by status of women’s employment^a^

	*Lower* status in women’s employment ^b^		*Higher* employment status women’s employment ^c^	
	OR	95% CI	OR	95% CI
*Individual-level variables*				
Caregiving	1.40	(0.87–2.26)	1.33^**^	(1.81–2.53)
Married	0.64^**^	(0.48–0.84)	0.39^**^	(0.25–0.63)
Employed	0.89	(0.66–1.21)	0.40^**^	(0.24–0.67)
Married x Employed	1.17	(0.84–1.64)	1.62^*^	(1.02–2.57)
Education	0.68^***^	(0.60–0.77)	0.84^*^	(0.75–0.95)
Age	1.03	(0.95–1.11)	0.97	(0.94–1.01)
N		8078		7022

^a^Status regarding women’s employment is measured by proportion of women employed in managerial or professional occupation, percent of female vulnerable employment, and rate of female labor force participation

^b^A higher status regarding women’s employment represents a higher proportion of women employed in managerial or professional occupation, a lower percent of female vulnerable employment, and a higher rate of labor force participation. These countries include USA, Israel, and those in Northern and Western Europe

^c^Countries with lower status regarding women’s employment are Southern Europe, Eastern Asia, and Mexico

* *p* < 0.05; **p < 0.01; ***p < 0.001

*Abbreviations*: *CI* confidence interval, *OR* odds ratio

As shown in Table 4, we have observed that caregiving has a negative effect in countries with lower rates of female labor force participation and higher adolescent fertility such as Mexico, Belgium, Spain, and Greece; this underscores caregiving as a deleterious factor in countries with a lower women’s status. In addition, caregiving was also found to be harmful in Germany and the UK, where the labor force participation rate and adolescent fertility rate are both higher. A higher labor force participation rate may be due to higher economic development in these countries, in other words a higher GNI, whereas women’s status may still remain relatively low in the reproductive domain.

**Table 4:** Prevalence of depressive symptoms (percentage) by country and caregiving status

*Country*	Caregiving status		
	No	Yes	*p*
People’s Republic of China	48.02	42.17	
Japan	19.49	18.71	
Korea	14.13	16.28	
Mexico	38.16	49.41	**
Taiwan	11.32	5.26	
UK	23.64	31.35	**
USA	19.24	15.46	
Austria	23.74	20.00	
Belgium	27.63	40.66	*
Denmark	25.19	20.00	
France	37.56	53.33	*
Germany	18.57	31.37	*
Greece	26.91	52.17	***
Israel	33.33	32.86	
Italy	34.94	51.72	
Netherlands	21.25	28.07	
Spain	33.33	64.71	**
Sweden	24.89	27.27	
Switzerland	28.28	25.00	

* *p* < 0.05; **p < 0.01; ***p < 0.001

By way of contrast, caregiving was not a significant or harmful factor in relation to depressive symptomatology in Japan, Korea, and Taiwan. One possible interpretation may go beyond the association between caregiving and the country-level indicators. Japan, Korea, and Taiwan share some similarities in historical background such as a Confucian ideology, previous Japanese administration, and a rapid industrialization during the twentieth century [11, 40]. This common tradition is a fundamental force that has shaped women’s economic roles and opportunities up to the present day [21, 40]. As a result, women’s status in these three East Asian countries may not have been raised to the same extent as other countries by economic growth, which is usually associated with greater female participation in economically productive work. Furthermore, there is a significant influence of Confucian patriarchal ideology on individuals in these countries and women are expected to provide care rather than being economically productive to their family. Even under the pressure of a capitalist economy, the influence of patriarchal beliefs still remains and a redefinition of care and work among East Asian women in a cultural context is warranted [41, 42]. Along these lines, the People’s Republic of China appears to be an interesting case. Although the People’s Republic of China would seem to have a common tradition with the countries mentioned above, there is a relatively higher rate of female labor force participation and of female vulnerable employment in China compared to other countries. This phenomenon is due to the financial necessities of dual wages for a family [42] as well as the People’s Republic of China’s employment policy in 1960s that was aimed at promoting gender equality through providing equal opportunities across the labor market [43]. This has led to a larger gender gap in power and resources [21, 44].

Our results substantiated the body of prior research that indicates the benefit of education for Baby Boomer women, as demonstrated by lower likelihoods of depressive symptoms among Baby Boomer women who have higher levels of education [45–47]. In addition, employment was found to be protective against depressive symptomatology among Baby Boomer women [21, 46]. Marital status, another factor related to social role, was also found to have a protective factor of depressive symptomatology. Furthermore, Baby Boomer women who are both employed and married were found to be significantly more likely to report depressive symptomatology than women who were neither employed nor married. As the *work-family conflict* model suggests [6, 48], the potential incompatibility between work and family responsibilities may produce women who have a greater spillover of family stress into work and thus a higher conflict level, which, in turn, is associated with an accumulation of stress and a higher odds of depressive symptomatology [5, 49].

Our findings should be interpreted within the context of the study’s limitations. In addition to the common limitations associated with self-reported measures, the association of individual-level and country-level factors with depressive symptomatology might be influenced by concerns regarding endogeneity. Baby Boomer women may not be randomly allocated to certain social roles and as caregivers for a number of reasons, including specific family backgrounds, social expectations, and a social status. Caregiving provision may also be influenced by social norms or specific country characteristics that have not been included in the analysis. When such factors are not completely taken into account, the allocation of variance to social roles and caregivers may result in a downward bias on the size of the country effects. In addition, instrumental caring in CHARLS was utilized as a proxy for personal care in the present study. In an analyses not shown here, we tested for the association between caregiving and depressive symptomatology, when the CHARLS survey was omitted. Notwithstanding the change, similar results were obtained. Cross-sectional data analyses are unable to disentangle or establish the causal links suggested herein. However, our analyses do show convincing associations between selected individual/country variables and depressive symptomatology. Our analyses are based on sets of multilevel logit models with random intercept and fixed coefficients only. Hence, our findings cannot provide evidence of the effects of individual-factor variance across countries. Nevertheless, we have conducted sensitivity analyses by standardizing CES-D scores rather than using cut-off points and the results regarding associations have remained robust.

## Conclusion

In summary, using a multilevel analytical approach, our study does provide important insights and is able to identify the social role component and some of its multilevel determinants with respect to depressive symptomatology around the world. The findings of this study parallel previous global research that has underscored the importance of educational attainment, employment, and marriage as independent protective factors and it also further highlights new insights regarding the role of being a caregiver and social class at country level as a risk factor among Baby Boomer women. Additional research is needed to gain a better understanding of depressive symptoms in a broader relationship context in order that policy makers can utilize this information to create and develop more effective prevention programs and organize interventions that promote mental health among Baby Boomer women and other similar demographic groups.

## Appendix

**Table 5:** Operational definitions of depressive symptoms across surveys

Dataset (year)	Measure of depressive symptoms	Range	Cut-off point	Reference
CHARLS (2011)	10 items CES-D	0–30	10	Andesen et al. (1994) [25]
JSTAR (2007)	20 items CES-D	0–30	16	Breslau (1985) [26]
KLoSA (2006)	10 items CES-D	0–30	10	Andesen et al. (1994) [25]
MHAS (2001)	9 items CES-D	0–9	5	García-Fabela et al. (2009) [27]
TLSA (2003)	10 items CES-D	0–30	10	Chang & Weng (2013) [28]
ELSA (2002)	8 items CES-D	0–8	3	Rice et al. (2009) [29]
HRS (2004)	8 items CES-D	0–8	4	Reyes-Gibby et al. (2006) [30]
SHARE (2004)	12 items EURO-D	0–12	4	Ladin et al. (2009) [31]

## Abbreviations

CHARLS: China Health and Retirement Longitudinal Study
ELSA: English Longitudinal Study of Aging
HRS: Health and Retirement Survey
JSTAR: Japanese Study of Aging and Retirement
KLoSA: Korean Longitudinal Study of Aging
MHAS: Mexican Health and Aging Study
SHARE: Survey of Health, Aging and Retirement in Europe
TLSA: Taiwan Longitudinal Study of Aging
